# Epigenetic Modifications and Their Role in Type 1 Diabetes Development: A Review

**DOI:** 10.2174/0115733998399336251122042738

**Published:** 2026-01-20

**Authors:** Jenner Chrystian Veríssimo de Azevedo, Fernando Liberalino Fernandes, Tayline Dantas Rodrigues, Iluska Almeida Carneiro Martins de Medeiros, Vania Sousa Andrade, Joselio Maria Galvão de Araújo, Fabiana Lima Bezerra, Christiane Medeiros Bezerra, Thales Allyrio Araújo de Medeiros Fernandes, José Veríssimo Fernandes

**Affiliations:** 1 Department of Pediatrics, Federal University of Rio Grande do Norte, 59078-970, Natal, Brazil;; 2 Department of Microbiology and Parasitology, Federal University of Rio Grande do Norte, Natal, 59078-970, Brazil;; 3 Department of Biomedical Sciences, Rio Grande do Norte State University, 59607-360, Mossoró, Brazil

**Keywords:** Type 1 diabetes mellitus, insulin deficiency, biomarkers, immune imbalance, RNA, pancreatic β-cell destruction, DNA methylation, metabolic disease

## Abstract

**Introduction:**

Type 1 Diabetes Mellitus (T1DM) is a multifactorial autoimmune disease marked by pancreatic β-cell destruction and insulin deficiency. Its pathogenesis involves genetic predisposition, environmental exposures, and epigenetic modifications. This review examines how epigenetic mechanisms, including DNA methylation, histone modifications, and non- coding RNAs, contribute to T1DM and their potential as biomarkers and therapeutic targets.

**Methods:**

A comprehensive literature review was conducted using PubMed, Scopus, Web of Knowledge, and Google Scholar. Studies on DNA methylation, histone modifications, and non- coding RNA expression in T1DM patients and experimental models were analyzed to identify mechanisms linking epigenetic mechanisms to disease progression.

**Results:**

Epigenetic alterations, including abnormal DNA methylation, histone modifications, and dysregulated non-coding RNAs, play central roles in immune imbalance and β-cell dysfunction. DNA methylation affects genes involved in immune regulation, insulin synthesis, and β-cell survival. Non-coding RNAs regulate transcriptional and inflammatory pathways, while histone modifications alter chromatin accessibility, further contributing to β-cell loss.

**Discussion:**

Epigenetic mechanisms mediate interactions between genetic risk and environmental triggers, shaping autoimmunity and disease heterogeneity. These findings underscore their relevance for early detection and targeted interventions in T1DM.

**Conclusion:**

Epigenetic changes form a critical link between genetics, environment, and immune dysfunction in T1DM. By regulating immune responses and β-cell integrity, they drive disease onset and progression. Their study provides opportunities for predictive biomarkers and innovative therapies aimed at reprogramming epigenetic pathways to restore immune tolerance and preserve β-cell function.

## 1 INTRODUCTION

Type 1 Diabetes Mellitus (T1DM) is an autoimmune disease linked to genetic, epigenetic, and environmental factors unique to each individual. It is characterized by hyperglycemia, polyuria, and inadequate insulin production due to pancreatic islet β-cell destruction by autoreactive immune cells [1, 2]. Dysregulation of the immune system, stemming from genetic, epigenetic, and environmental factors, leads to autoimmunity, with insulitis playing a key role [2, 3].

Historically, the acquired immune system has been distinguished from the innate immune system by its specificity and memory. However, recent findings challenge this notion, revealing that innate immune cells can develop a memory phenotype termed “trained immunity” [4]. Trained innate immunity is associated with metabolic memory, where aerobic glycolysis provides energy for heightened immune cell activation [5]. Elevated glucose levels in T1DM patients may trigger metabolic memory, which may potentially exacerbate late vascular complications [6].

The mechanism of trained innate immunity involves activating the mTOR-HIF-1α pathway and reprogramming innate immune cells *via* histone modifications, enhancing gene transcription upon re-stimulation [4, 5]. Epigenetic mechanisms are crucial in an organism's evolutionary process, protecting against stressors and ensuring survival [2, 7]. However, in chronic inflammation, they can worsen hyperinflammation and advance autoimmune diseases by increasing pro-inflammatory cytokine production, causing tissue damage, including in pancreatic islets [4, 8].

Epigenetic modifications alter cellular genetic function, and they are capable of transmitting changes across generations without altering the DNA sequence. They regulate gene expression through DNA chemical modifications, accessibility, or chromatin structure [9, 10]. Reversible yet potent, these modifications can induce gene overexpression or silencing, resulting in phenotypic changes independent of genotype alterations [2, 11, 12]. Notable mechanisms of epigenetic inheritance include cellular reprogramming, chromatin remodeling, histone modifications, DNA modifications, non-coding RNA expression, and alternative mRNA splicing, all influencing chromatin structure and promoter accessibility, ultimately leading to gene silencing or activation [13].

Epigenetic modifications affect metabolic and molecular pathways, changing gene expression and impacting health [14]. Epigenetic regulation plays a crucial role in immune system function and β-cell integrity. Dysregulation, influenced by external factors, can modify gene expression by altering transcription factors, signaling pathways, or enzymes, thereby contributing to the development of Type 1 Diabetes (T1DM) [15]. Environmental stress, lifestyle factors, and exposure to pollutants trigger epigenetic mechanisms that foster autoimmune diseases such as T1DM [14].

The etiopathogenesis of T1DM is multifaceted, involving immune system dysregulation and breakdown of self-tolerance, leading to autoimmunity. This complex process is influenced by genetic, epigenetic, and environmental factors, disrupting organismal homeostasis [16]. Recent evidence indicates that T1DM onset is not solely immune-driven but also involves dysfunction and/or death of pancreatic islet β- cells due to metabolic stress overload [17]. The interplay between immune cells and β-cells appears crucial, precipitating insulitis before T1DM onset [18]. This review presents recent advances on the role of epigenetic modifications in mechanisms that lead to the breakdown of immunological self-tolerance, thereby triggering the autoimmune response that culminates in the initiation and progression of the disease.

## 2 METHODS

The literature review presented in this study was conducted using PubMed (National Institutes of Health; ncbi.nlm.nih.gov/pubmed), Scopus (Elsevier; scopus.com/scopus/home.url), and Web of Knowledge (Thomson Reuters; wok. mimas.ac.uk), as well as Google Scholar. The search utilized the following keywords: 'Type 1 diabetes', 'Breakage of self- tolerance', 'Autoimmunity', 'Metabolic disease', 'Destruction of pancreatic β-cells', 'Pathogenesis of type 1 diabetes', 'Immune system dysfunction in type 1 diabetes', 'β-cell dysfunction in type 1 diabetes', 'Role of β-cells in activating the autoimmune response in type 1 diabetes', 'Role of epigenetic modifications in type 1 diabetes', 'DNA methylation in type 1 diabetes', 'Histone modifications in type 1 diabetes', 'Role of non-coding RNAs in type 1 diabetes'.

Several hundred articles were retrieved from the databases searched, with only the most relevant ones selected. These articles were published in journals with a high impact factor and authored by groups recognized for their expertise in the field.

## 3 PATHOGENESIS OF TYPE 1 DIABETES

The pathogenesis of type 1 diabetes (DM1) involves the action of multiple factors that interact with each other through a complex network of events, resulting in an organ-specific autoimmune disease. In this condition, CD8+ T cells and other autoreactive immune cells target and destroy pancreatic β-cells, which are responsible for producing insulin within the islets of Langerhans. These autoimmune responses are triggered by peptides produced by pancreatic islet β-cells, which are presented in association with class I molecules of the Major Histocompatibility Complex (MHC). These peptides are generated in the cytoplasm of β-cells through the proteasomal degradation of endogenous proteins, after which they bind to MHC I molecules and are transported to the cell surface [19, 20].

Among the autoantigens involved are insulin, glutamic acid decarboxylase (GAD), proteins related to the catalytic subunit of islet-specific glucose-6-phosphatase, islet-associated amyloid polypeptide, and post-translationally modified peptides or neoantigens [21, 22]. In type 1 diabetes (DM1), the autoimmune response occurs when effector T lymphocytes recognize self-antigens from β-cells and are not suppressed by regulatory T cells (Tregs) due to a failure in peripheral tolerance [17]. Treg cells function to suppress the activation and effector action of autoreactive T cells activated in the periphery. There is strong evidence that this specific function of Treg cells is impaired in T1D [23].

The breakdown of self-tolerance begins in the thymus during the development of the T cell repertoire, wherein CD4+, CD8+ lymphocytes, and natural Treg cells (CD4+, CD25, FoxP3+) are formed. Throughout lymphocyte maturation, central tolerance aims to eliminate autoreactive cells and form FoxP3+ Treg cells, which suppress autoimmune cells in the periphery. However, failure in negative selection may lead to the maturation of specific CD4+ and CD8+ lymphocytes against self-antigens, such as those from pancreatic β-cells. Moreover, the formation of β-cell-specific FOXP3+ Treg cells may occur inefficiently, resulting in Treg cells with reduced ability to suppress autoreactive T cells in the periphery. After maturation in the thymus, both naïve T lymphocytes and FOXP3+ Treg cells specific for β-cell autoantigens migrate to the periphery and recirculate through peripheral lymphoid organs [24].

Various triggers can initiate the autoimmune response against β-cells, including infection, inflammation, and metabolic stress [25, 26]. Under these conditions, dendritic cells in the pancreas capture antigens from β-cells and migrate to pancreatic lymph nodes, where they process and present them to CD4+ and CD8+ T lymphocytes. Antigens processed *via* the endocytic route associate with MHC class II and are presented to CD4+ T lymphocytes, while those processed *via* the cytosolic route bind to MHC class I and are presented to CD8+ T lymphocytes. In the lymph nodes, the activation and differentiation of autoreactive effector cytotoxic Th and T lymphocytes occur, which migrate to the pancreatic islets. When the suppressive function of Treg cells fails, the autoimmune response against β-cells is triggered by self-peptides associated with MHC molecules [27].

In insulitis, which results in the destruction of pancreatic islet β-cells, cytotoxic CD8+ T lymphocytes predominate among infiltrating immune cells [28]. The inflammatory cytokines produced in this environment cause the overexpression of MHC class I molecules, increasing the presentation of self-antigens produced by β-cells to effector CD8+ T lymphocytes [29, 30]. Cytotoxic T cells, in turn, induce the death of β-cells by apoptosis through the Fas-FasL interaction and the action of perforin and granzyme-B [31, 32].

Recent studies demonstrate that CD8+ T cells specific for β-cells in T1DM maintain their autoreactive potential despite continuous access to the antigen [33, 34]. These CD8+ T cells behave like autoimmune progenitors akin to stem cells, self-renewing and giving rise to effector cells, exhibiting a pattern of epigenetic multipotency similar to stem cells. Thus, the differentiation status of T cells is regulated by stable epigenetic modifications in DNA methylation patterns, enabling the cell to acquire a profile that is simultaneously naïve and effector. This mechanism explains why β- cell-specific CD8+ T cells maintain a stem cell-like status to sustain long-lasting active immune responses despite constant antigen exposure [33].

Figure (**1**) represents a schematic summary of the pathogenesis of T1DM, illustrating the main mechanisms leading to dysregulation of the immune system and the dysfunction **or** death of pancreatic islet β-cells, ultimately resulting in the autoimmune process that leads to the destruction of pancreatic islet β-cells and the development of the disease (Fig. **1**).

### 3.1 Epigenetic Mechanisms

It has been proposed that inflammation of the pancreatic islets with infiltration of immune cells, known as insulitis, is a crucial element in the pathogenesis of T1DM, and that other factors are necessary for the initiation and progression of the disease. Among the numerous factors involved in this process, epigenetically induced changes in gene expression triggered by environmental factors seem to play a critical role.

Epigenetic modifications can be categorized into three main groups: DNA methylation, histone modifications, and regulation of non-coding RNAs (ncRNAs) [10, 35]. DNA methylation or acetylation can lead to post-translational modifications of histones, which can also occur due to dysregulation of ncRNAs, triggered by exogenous stimuli and environmental factors that regulate cell differentiation and organ development [9, 36]. Gene expression regulation within the human body is orchestrated through the activation or inhibition of signaling pathways and the action of molecules that interact with DNA, which can contribute to health or disease [37, 38]. This process is mediated by the epigenetic machinery that governs gene expression in eukaryotes, involving a complex interplay among over 600 epigenetic regulatory genes that read, write, and erase histone and DNA modifications [39].

Epigenetic mechanisms, essential for cellular development and function, also play a role in the onset and progression of complex diseases such as T1DM, which arises from the interaction between genetic predisposition and environmental exposures. Polymorphisms identified through genome-wide association studies (GWAS), particularly in genes within the major histocompatibility complex (MHC), contribute to disease susceptibility [40]. Environmental factors, such as diet, viral infections, and alterations in the gut microbiota, modulate this risk *via* epigenetic mechanisms, primarily through changes in DNA methylation and histone acetylation. Thus, epigenetics serves as a link between the environment and the genome, regulating genes involved in immune response and both central and peripheral tolerance [41].

#### 3.1.1 DNA Methylation

Methylation is the process by which a hydrogen atom is replaced by a methyl group *via* a covalent bond at the 5th carbon position of the cytosine nucleotide, preceding the guanine nucleotide (CpG), catalyzed by DNA methyltransferase enzymes (DNMT). These sites on the DNA strand where these reactions occur are termed CpG methylation islands [42]. While most CpG islands in the mammalian genome are methylated, the effect of DNA methylation on regulating gene expression depends on its position within the gene. Methylation within the gene body diminishes RNA polymerase's ability to bind and initiate transcription; promoter methylation is generally associated with gene silencing [43]. Methylation of DNA CpG islands and/or their hydroxymethylation is one of the epigenetic events associated with the pathophysiology of autoimmune inflammatory diseases, resulting from the interplay of genetic susceptibility and epigenetic modifications induced by environmental exposure [44].

In a study examining the progression of T1DM in the NOD mouse model, an inverse relationship between the proportion of infiltrating lymphocytes and the mass of insulin-producing β-cells was observed. Additionally, methylation marks of insulin-encoding genes (Ins1 and Ins2) changed over time. The expression of insulin genes appears to be more tightly downregulated by methylation of Ins1 exon 2 and Ins2 exon 1. The transcription levels of cytokine genes increased with the age of the animals, and these cytokines can induce methylation of the promoters of insulin-coding genes through the action of methyltransferase enzymes. Cytokines in human β-cells induced similar changes *in vitro*. This demonstrates that epigenetic modification by DNA methylation in β-cells exposed to immunological stress can contribute to the reduction of insulin production and, consequently, to the progression of T1DM [45].

Analysis of DNA methylation in blood cells of participants in the longitudinal Diabetes Complications Screening and Control (DCCT) study revealed that long-term hyperglycemia increased DNA methylation levels and was associated with elevated mean glycated hemoglobin (HbA1c) levels. The persistence of DNA methylation patterns in monocytes collected 16 or 17 years after the onset of the disease, as observed in the Epidemiology of Diabetes Complications and Interventions Study (EDIC), suggests the presence of metabolic memory [46]. HbA1c levels and the time required for T1DM to develop were positively correlated with age and DNA methylation levels [47]. Differences in DNA methylation patterns in both blood and saliva have been observed between individuals with T1DM and end-stage renal disease and those without renal disease [48-50].

In a case-control study, higher levels of CpG island methylation were observed in the case group, with 19 sites associated with the risk of developing diabetic nephropathy [51]. Furthermore, higher DNA methylation values were associated with decreased kidney function [47]. Analysis of genome-wide DNA methylation levels in blood samples from individuals with T1DM identified 349 sites, corresponding to 233 genes, that were differentially methylated between individuals with proliferative diabetic retinopathy and those with long-term diabetes without retinopathy or with mild retinopathy [52].

A study involving 778 Swedish individuals, including T1DM carriers with or without diabetic nephropathy (DN) and individuals with Normal Glucose Tolerance (NGT), analyzed methylation levels of the IGFBP1 gene in peripheral blood cells and the expression levels of this gene. It was hypothesized that the IGFBP1 gene could play a potentially beneficial role in regulating metabolic and vascular homeostasis. IGFBP1 methylation levels were reduced, while its serum levels were increased in T1DM patients compared with NGT controls. Furthermore, T1DM patients with DN had higher serum IGFBP-1 concentrations compared to patients without DN. However, no difference in IGFBP1 methylation levels was observed between T1DM patients with or without DN. It was concluded that patients with T1DM exhibit decreased methylation of the IGFBP1 gene and that elevated serum IGFBP-1 levels are associated with the disease [53].

A genome-wide association study (GWAS) analyzed DNA methylation profiles in lymphocytes from three pairs of hemizygous twins discordant for T1DM and six pairs of hemizygous twins concordant for the disease, using the HumanMethylation450 BeadChip. The methylation status of 27,578 CpG islands, located mainly in proximal promoter regions, was evaluated. Eighty-eight CpG islands exhibiting changes in DNA methylation levels were identified in all twins with T1DM. Functional annotation of genes with altered methylation in individuals with T1DM showed that they regulate immune response pathways and are associated with T1DM, such as HLA, INS, IL-2RB, and CD226, which exhibit significantly higher DNA methylation levels in twins with T1DM compared to non-diabetic twins. Abnormal DNA methylation patterns in diabetics suggest that this epigenetic change may be involved in the pathogenesis of the disease [54].

Epigenomic studies have shown that immune cells involved in the pathogenesis of T1DM exhibit distinct epigenetic profiles. Autoreactive CD8^+^ T lymphocytes, specific for pancreatic β-cell antigens, display a “stemness” pattern characterized by plasticity and prolonged persistence, promoting chronic cytotoxic activity and sustaining autoimmunity [33]. Regulatory T cells (Tregs), essential for immune homeostasis, exhibit hypermethylation of the FOXP3 and IL2RA promoters, which is associated with impaired immunosuppressive function and immune regulatory failure [41, 55].

This epigenetic imbalance between effector and regulatory cells creates an immune environment marked by persistent inflammation and defective immune regulation. Emerging technologies, such as single-cell epigenomics, have enabled the identification of these alterations in specific T cell subpopulations, deepening the understanding of the molecular mechanisms underlying autoimmunity in T1DM. These advances pave the way for epigenetic reprogramming-based therapies with the potential to restore immune tolerance [56].

One study analyzed 52 pairs of homozygous twins discordant for T1DM for DNA methylation across the entire genome in three immune cell subsets: CD19+B cells, CD14+ monocytes, and CD4+ T cells. CD14+ monocytes and CD4+ T cells from 12 twin pairs discordant for T1DM, including 201 individuals with T1DM and 139 healthy, unrelated individuals, and umbilical cord blood from 98 newborns, of which 50 progressed to clinically evident T1DM during infancy. Characterization of differentially DNA methylated Variable Positions (DVPs) associated with the disease was also conducted using gene regulatory circuits specific to each cell type. Differential DNA methylation, determined by two methods, was higher in twins with T1DM than in their healthy co-twins and in healthy, unrelated individuals. It was observed that methylation of CpG islands associated with T1DM is stable over time and is richer in gene regulatory elements. Among the gene regulatory circuits specific to each cell type, pathways related to metabolism and the cell cycle stand out, including the mTOR signaling pathway. Evidence was found in the umbilical cord blood of the 50 newborns who progressed to T1DM that VPDs emerge after birth. The findings indicate that epigenetic changes, including methylation, may contribute to the pathogenesis of the disease [57].

A longitudinal case-control study followed 2,547 children from Colorado, USA, with a high genetic risk for T1DM from birth. Blood samples were initially collected at 9, 15, and 24 months of age, and then annually until the emergence of insulin autoantibodies. Subsequently, individuals who tested positive had their blood drawn every 3 to 6 months until diabetes was diagnosed. Of the participants who presented with autoantibodies, 87 who developed the disease were considered cases and were paired with an equal number of individuals who had not developed the disease as controls up to that point. Subsequently, the DNA methylation patterns of the two groups were analyzed in the blood samples collected throughout the study. The average number of methylations differed between cases and controls at two positions and 28 genomic regions. Some methylation differences were detectable and consistent from birth, both before and after the onset of preclinical symptoms. Changes in methylation with age were detected, differing between cases and controls in 10 genomic regions. The methylated genes included both transcription factor coding and non-coding genes. Methylation differences between cases were observed both before the appearance of islet autoantibodies and after the clinical diagnosis of T1DM, indicating that this epigenetic change plays a role in the development of the disease [58].

A meta-analysis evaluated differences in genome-wide DNA methylation levels in blood cells from 1,304 carefully characterized individuals with T1DM and known renal status across two cohorts: one from the Republic of Ireland and the United Kingdom, and the other from Finland. Thirty-two differentially methylated CpG islands were identified in T1DM carriers with diabetic kidney disease, 18 of which were in genes differentially expressed in the kidney or correlated with pathological features in diabetic kidney disease. It has been proposed that methylation at 21 of the 32 identified CpG islands may predict the development of renal failure, allowing the identification of individuals at increased risk of developing diabetic kidney disease in T1DM [59].

In a case-control study, 87 individuals with T1DM and 87 healthy controls were analyzed for the causal relationship between DNA methylation in 12 CpG islands of the DLGAP2 gene and a family history of the disease. The risk of T1DM was estimated using two logistic regression models adjusted for HLA genotype DR3/4. A significant association was identified between family history of T1DM and DNA methylation at 8 of the 12 CpGs analyzed, indicating a role for DNA methylation in the risk for the disease. It was found that children of diabetic mothers had a higher risk of developing the disease compared to those without a family history. This increased risk was mediated by differences in the methylation levels of the CpG islands (cg27351978 and cg00565786) in the DLGAP2 gene. In contrast, the risk for daughters of diabetic parents to develop T1DM did not correlate with methylation of the two CpG islands, suggesting a maternal protective effect against in utero T1DM exposure that reduces DNA methylation [60].

A longitudinal genome-wide association study (GWAS) of DNA methylation, carried out over 28 years in blood cells, analyzed 683,597 CpGs from an observational cohort of T1D patients. The longitudinal analysis revealed hypomethylation of the TXNIP gene (cg19693031) correlated with increased glycated hemoglobin levels. This gene encodes the protein thioredoxin, which plays an important role in regulating glucose and lipid metabolism, including β-cell function. High TXNIP expression was consistently associated with elevated glycated hemoglobin levels over 28 years of patient follow-up. Furthermore, pulse rate, triglycerides, and albumin/creatinine ratio remained elevated, regardless of glycated hemoglobin. This indicates the therapeutic potential of interventions targeting the TXNIP gene to improve glycemic control in T1DM [61].

Evidence indicates epigenetic alterations in renal genes associated with diabetic nephropathy. Genes involved in inflammatory, fibrotic, and apoptotic processes, like RASAL1, are epigenetically regulated. Its promoter's hypermethylation activates the Ras-GTP pathway, promoting renal fibrosis [62]. Similar changes occur in mTOR, NPHS1, and MIOX, related to oxidative stress, podocyte loss, and increased albuminuria [63]. MicroRNAs such as miR-21, miR-192, and miR-377, overexpressed in diabetic renal tissue, contribute to mesangial hypertrophy and extracellular matrix accumulation [64]. Detected in urinary exosomes, these miRNAs are proposed as biomarkers for early diagnosis and nephropathy monitoring [65].

Similarly, diabetic retinopathy involves epigenetic changes affecting vascular and neural retina function. Global DNA methylation and modifications in mitochondrial genes, such as POLG, impair redox homeostasis and increase retinal oxidative stress [66]. Histone methylation, especially H3K9me by SUV39H2, activates inflammatory pathways, such as NF-κB, and upregulates mediators, such as MMP-9. These mechanisms cause endothelial dysfunction, increased vascular permeability, and abnormal angiogenesis, key events in proliferative retinopathy [67]. Circulating miRNAs such as miR-126, miR-210, and miR-150-5p are linked to retinopathy progression [64].

Monitoring epigenetic signatures, including methylation profiles of renal/retinal genes and quantification of circulating miRNAs, has the potential to stratify risk, facilitate early diagnosis, and predict the prognosis of diabetic complications [68]. Epigenetic therapies, such as DNMT and HDAC inhibitors, show efficacy in experimental models, offering promising therapeutic approaches [63].

The role of DNA methylation in diabetic nephropathy (DN) was analyzed in leukocytes from participants enrolled in the Finnish T1DM study (FinnDiane). Initially analyzed in 39 individuals and later expanded to 296, it was found that the body of genes accounted for more than 60% of the differential methylation. The differentially methylated genes associated with DN were then analyzed in three independent groups of individuals with T1DM: 445 from Denmark, 107 from Hong Kong, and 130 from Thailand. DNA methylation was reduced in the CTCF and Pol2B genes and was closely linked to DN pathways, including insulin signaling, lipid metabolism, and fibrosis. To define the pathophysiological significance of the findings, DNA methylation indices were evaluated in human kidney cells, such as podocytes and proximal convoluted tubule cells. High expression of CTCF and Pol2B genes was associated with hypomethylation and reduction of insulin signaling proteins in hyperglycemic cells. Similar results were observed in methylation-mediated regulation in human macrophages and vascular endothelial cells [69].

A cohort study of 25 children recently diagnosed with T1DM and 25 healthy controls showed that the rs689 Single Nucleotide Polymorphism (SNP) is associated with methylation in four CpG islands of the INS gene promoter. All of them are related to the AA genotype, which is associated with susceptibility to T1DM and hypermethylation compared to other genotypes. It was also observed that the rs12722495 site of the IL2RA gene promoter in B cells with the AA risk genotype for T1DM is hypomethylated compared to that of the AG genotype. Comparison of methylation levels between individuals with T1DM and healthy controls showed significant differences in INS gene promoter methylation in CD8+ T cells from children with DM1, suggesting an association with the disease and the immune response. Taken together, these results suggest that the genetic effect caused by DNA methylation contributes, at least in part, to the pathogenesis of T1DM [70]. In Fig. (**2**), genes are depicted whose expression is altered in individuals with T1DM due to changes in DNA methylation, characterized by both hypermethylation and hypomethylation.

#### 3.1.2 Histone Modifications

Histones are globular proteins that assist in the orderly packaging of DNA within the cell nucleus, and they are responsible for regulating gene expression within the cell [71]. Accumulating evidence indicates that aberrant histone modification profiles are associated with dysregulation of the immune response, contributing to the pathogenesis of various autoimmune diseases [72]. Studies demonstrate the role of regulatory epigenetic mechanisms in the normal differentiation and function of the pancreas, and alterations in these mechanisms lead to metabolic diseases such as diabetes. In this context, histone modifications mediate gene expression by regulating interactions of transcription factors and their target genes, thereby defining the fate of pancreatic cell differentiation. Consequently, histone modifications may trigger mechanisms that lead to metabolic diseases, including T1DM [71, 73].

Aberrant epigenetic changes, including histone modifications, may contribute to the pathogenesis of T1D by regulating the expression of disease-related susceptible genes. Notable among these genes are those of the HLA system, associated with antigen presentation, which are positively regulated; those related to immunological tolerance, such as FOXP3 and CTLA4, which are negatively regulated; those related to the autoreactive T cell response, such as GAD65, which is upregulated; and those involved in β-cell functions, such as INS, which is downregulated [74, 75]. Histone modifications by acetylation and methylation appear to play a crucial role in the pathogenesis of diabetic kidney disease, affecting several molecular pathways involved in the induction of inflammation, oxidative stress, and fibrosis in the kidneys of diabetic patients [76].

#### 3.1.3 Non-coding RNAs

Non-coding RNAs (ncRNAs) are RNA molecules that are not translated into proteins. The mammalian genome encodes several types of these transcripts, which have multiple regulatory functions [77]. The main classes of ncRNAs are microRNAs (miRNAs), long non-coding RNAs (lncRNAs), and circular non-coding RNAs (circRNAs) [78]. miRNAs are 22-nucleotide endogenous RNAs that act as key regulatory molecules in the expression of genes involved in multiple pathways and cellular systems. Both long and circular non- coding RNAs are composed of more than 200 nucleotides. Many of these RNAs play crucial roles in critical biological processes, through epigenetic control of chromatin, promoter-specific gene regulation, and mRNA stability [77, 79].

NcRNAs represent a diverse set of molecules that regulate gene expression and fundamental physiological processes for the homeostasis of the human organism. They regulate cell division and differentiation, proliferation, apoptosis, metabolism, and the functioning of the immune system [79]. A growing body of evidence points to a critical role of ncRNAs in the initiation and progression of T1DM. Furthermore, due to their importance in maintaining physiological homeostasis by regulating gene expression, they can serve as biomarkers for diagnosis and as potential therapeutic targets in the treatment of the disease [78].

A case-control study measured serum levels of 745 miRNAs in children with new-onset T1DM and in age-matched controls, using enhanced quantitative PCR. Thirty-five miRNAs were found with significant differences in expression levels in the two groups, of which 27 were elevated in T1DM. Among these, six: miR-454-3p, miR-222-3p, miR-144-5p, miR-345-5p, miR-24-3p, and miR-140-5p, had expression levels similar to those of controls up to six months after diagnosis of the disease. Bioinformatic analysis, which identified the target genes of these miRNAs, suggests an association with the biosynthesis of glycosaminoglycans and with the PI3K/Akt, MAPK, and Wnt signaling pathways, all involved in the early stages of T1DM. Of the 27 miRNAs upregulated in T1DM, two of them, miR-324-5p and let-7e-5p, were negatively correlated with glycated hemoglobin. Of the eight miRNAs that were downregulated, five: miR-154, miR-490-5p, miR-630, miR-639, and miR-675 were positively associated with glycated hemoglobin [80].

miR-101-3p and miR-204-5p are highly expressed in pancreatic β-cells, where they negatively regulate insulin secretion and cell survival, and their roles in the pathogenesis of T1DM were investigated. Serum levels of miR-101-3p and miR-204-5p were analyzed in four groups: (1) patients with new-onset T1DM, (2) individuals with normal glucose levels but with islet autoantibodies, (3) individuals with normal glucose levels but with multiple types of autoantibodies, and (4) healthy subjects as controls. The relative expression of serum miR-101-3p was approximately 3 times higher in individuals with T1DM and multiple autoantibodies compared to controls. No significant differences in miR-204-5p expression were observed between the groups analyzed. Bioinformatics analysis of miR-101-3p target gene pathways indicated potential activation of HGF/c-Met, Ephrin receptor, and STAT3 signaling pathways. This indicates that miR-101-3p expression precedes the loss of glucose homeostasis and that its pathogenic role may involve multiple molecular pathways [81]. In Table **1**, several microRNAs that are upregulated in various stages of T1DM evolution are presented.

miRNA-146a-5p is considered a key regulator of inflammatory processes, and its expression is altered in target organs in diabetic complications. The reduced expression of this transcript has been implicated in the pathogenesis of T1DM. A case-control study of a large cohort investigated miRNA-146a-5p expression levels in patients with T1DM and healthy controls and found significantly lower levels in patients with T1DM. It was found that serum levels of this transcript were inversely associated with all chronic complications of the disease, particularly cardiovascular diseases [82].

A study carried out in obese mice identified an lncRNA (βFaar), enriched in pancreatic islets, which was shown to play a role in β-cell dysfunction, acting as a negative regulator of apoptosis. βFaar is drastically downregulated in the islets of obese mice, and this condition is necessary to cause obesity-associated β-cell dysfunction and apoptosis. βFaar has been shown to promote insulin synthesis and secretion by upregulating islet genes Ins2, NeuroD1, and Creb1, functioning as a sponge to inhibit the function of miR-138-5p. The TRAF3IP2 and SMURF1 proteins were identified as interactive factors associated with βFaar function. The βFaar gene inhibits the activity of the SMURF1 ubiquitin ligase, which is essential for TRAF3IP2 ubiquitination and activation of NF-κB-mediated β-cell apoptosis [83].

The role of microRNAs (miRNAs) in the onset and progression of Type 1 Diabetes Mellitus (T1DM) was assessed by evaluating 377 of these transcripts in serum samples from 110 patients categorized into four groups: healthy individuals (control), patients with only autoantibodies, patients with recent-onset T1DM, and patients with long-standing T1DM. A total of 69 miRNAs were found to be differentially expressed in patients with T1DM compared to controls. Several miRNAs exhibited correlations with islet autoantibodies (IA2A, GADA, and Znt8A), age, and elevated C-peptide levels, particularly in patients with recent-onset T1DM, suggesting their relevance in disease initiation and progression. Numerous miRNAs have been associated with metabolic disorders, inflammatory pathways, and autoimmune diseases. The most upregulated miRNAs were miR-200a-3p in individuals with autoantibodies or recent disease, and miR-346 in those with long-standing disease, while miR-16-3p was the only downregulated miRNA across all groups. The target of miR-200a-3p is the PRPP gene, which is associated with metabolic pathways, while miR-16-3p targets the GADD45 gene, which is involved in cell cycle control, senescence, and autophagy pathways [84].

One study employed high-throughput sequencing to analyze samples from patients with T1DM and individuals with normal glucose tolerance serving as controls. Forty-one miRNAs were identified as differentially expressed in patients with T1DM compared to controls, with a focus on miR-21-5p, which had the highest number of predicted target genes and was associated with various pathways, including insulin signaling and apoptosis. Five other miRNAs (miR-26b-5p, let-7i-5p, miR-143-3p, miR-501-3p, and miR-100-5p) were assessed using RT-qPCR to identify target genes. Among these, miR-26b-5p, miR-100-5p, and miR-143-3p were highly expressed and targeted genes encoding the insulin-like growth factor receptor (IGF1R), which plays a crucial role in the regulatory network of T1DM and influences numerous metabolic pathways, potentially including nuclear and mitochondrial dysfunction. Notably, miR-26b-5p regulates both IGF1R and IGF1, genes related to insulin signaling and apoptotic events [85].

Another study aimed to identify genes abnormally expressed in peripheral blood mononuclear cells of patients with T1DM to understand the regulatory molecular network of these genes. Among the 216 differentially expressed and functionally related genes directly or indirectly associated with T1DM, pathways such as cytotoxicity mediated by natural killer cells, Th1 and Th2 differentiation, and IL-17 and TNF signaling were implicated. The miRNA/mRNA and transcription factor network suggested that miR-320a and the transcription factor SOX5 induce Th17 cell differentiation and are the only ones targeting the ADM gene, which has diverse functions, including promoting vasodilation and angiogenesis, and the RRAGD gene, which is involved in the negative regulation of autophagy [86]. Table **2** presents several microRNAs that are downregulated at different stages of T1DM progression.

Exosomes, multienzyme protein complexes involved in RNA processing and degradation, carry various transcripts, including non-coding RNAs (ncRNAs), and play a vital role in cell-to-cell communication, influencing multiple physiological and pathological processes. Analysis of exosomal mRNA expression profiles in the plasma of 40 patients with T1DM and 40 age- and sex-matched controls using the Illumina HiSeq platform identified 112 differentially expressed mRNAs, including 66 upregulated and 46 downregulated mRNAs [87].

Long non-coding RNA (lncRNA) expression profiles were analyzed by real-time quantitative PCR (qRT-PCR) on the Illumina HiSeq platform from plasma samples of 30 individuals with T1DM and 30 healthy controls matched for age and sex. Among the 162 exosomal lncRNAs found to be differentially expressed in patients with T1DM, 77 were upregulated and 85 were downregulated. In contrast, only 77 lncRNAs were differentially expressed in healthy individuals [88].

Another study investigated the role of abnormal expression of lncRNAs PVT1 and LINC00960 in the development of T1DM and its complications. These lncRNAs were found to be involved in insulin secretion pathways by pancreatic islet β-cells, affecting the expression of genes such as Hippo, TGF-beta, Wnt, FOXO, Neurotrophin, and ErbB, through competitive regulation mediated by miR-107. It has been suggested that these two lncRNAs may contribute to the inflammatory response in T1DM [89].

A study evaluated the profiles of circular RNAs (circRNAs) expressed in CD4+ T cells from patients with T1DM and healthy volunteers. A total of 257 differentially expressed circRNAs were identified in patients with T1DM, with only three being upregulated (hsa_circ_0000324, hsa_circ_0001853, and hsa_circ_0068797). KEGG analysis revealed that these circRNAs were associated with 11 miRNAs and 8 mRNA target genes related to the immune system. Further analysis showed that four miRNAs, two circRNAs, and four mRNAs were associated with nine circRNA/miRNA/mRNA networks, wherein circRNAs act as sponges to inhibit miRNA function. qRT-PCR analysis suggested that circRNA000324/miRNA675-5p/MAPK14 and circRNA000324/miRNA-675-5p/SYK may be potential mechanisms in regulating CD4+ T cell proliferation in T1D patients [90].

A case-control study analyzed plasma samples from 20 patients with T1DM who were fasting and 10 healthy individuals. Next-generation sequencing was performed to identify miRNAs differentially expressed between the two groups. The miR-1-3p, miR-200b-3p, and miR-9-5p were identified. Bioinformatics analysis revealed the main pathways affected by the target genes of these miRNAs. The expression of miR-1-3p was significantly increased in T1DM patients compared to controls and was positively correlated with elevated glycated hemoglobin levels. It was observed that changes in miR-1-3p expression directly affected KEGG genes involved in pathways related to cardiovascular pathologies, endocrine disorders, inflammatory, and immunological diseases [91].

Another case-control study involved 12 adolescents aged 15.0-17.9 years with T1DM lasting more than 8 years (mean 11.1 years), enrolled in the Swedish Diabetes Quality Register, and a group of healthy individuals of the same age as the controls. Quantitative PCR analysis identified 187 circulating miRNAs, of which 27 were upregulated and one was downregulated in the T1DM group. Six miRNAs were enriched in blood, gastrointestinal, nervous, and thyroid tissues. Another six, with greater differences in plasma, of which five were positively regulated (miR-101-3p, miR-135a-5p, miR-143-3p, miR-223-3p, and miR-410-3p), and one was negatively regulated (miR-495-3p) in patients with T1DM, with P values below 0.01, were selected for in-silico analysis, which identified AKT1, VEGFA, and IGF-1 genes as common targets [92].

## CONCLUSION

The etiopathogenesis of Type 1 Diabetes Mellitus (T1DM) involves genetic predisposition and environmental factors, including infections, the microbiota, and epigenetic mechanisms. Metabolic stress in pancreatic β-cells triggers gene expression changes, increasing antigen presentation and activating stress pathways that lead to β-cell apoptosis or autophagy. Immune dysregulation further promotes β-cell destruction, impairing insulin production.

Epigenetic modifications contribute to T1DM by regulating autoreactive CD8+ T cells and protective regulatory T cells, notably through DNA methylation, non-coding RNAs, and histone modifications. These changes affect key genes involved in metabolism and immune tolerance.

Although progress has been made, the precise epigenetic mechanisms in T1DM remain incompletely understood. Further research is essential to identify epigenetic targets for biomarkers and personalized therapeutic approaches.

## AUTHORS’ CONTRIBUTIONS

The authors confirm contribution to the paper as follows: J.C.V.A.: Involved in the conception of the study, the literature review, and the drafting of the manuscript; F.L.F.: Involved in drafting the manuscript; T.D.R.: Involved in the critical revision of the manuscript for important intellectual content; I.A.C.M.M., V.S.A., and J.M.G.A.: Involved in the literature review; F.L.B.: Involved in revising the manuscript critically for important intellectual content; T.A.A.M.F.: Involved in revising the manuscript critically for important intellectual content; J.V.F.: Involved in the conception of the study, literature review, and drafting the manuscript. All authors read and approved the final manuscript.

## Figures and Tables

**Fig. (1):**
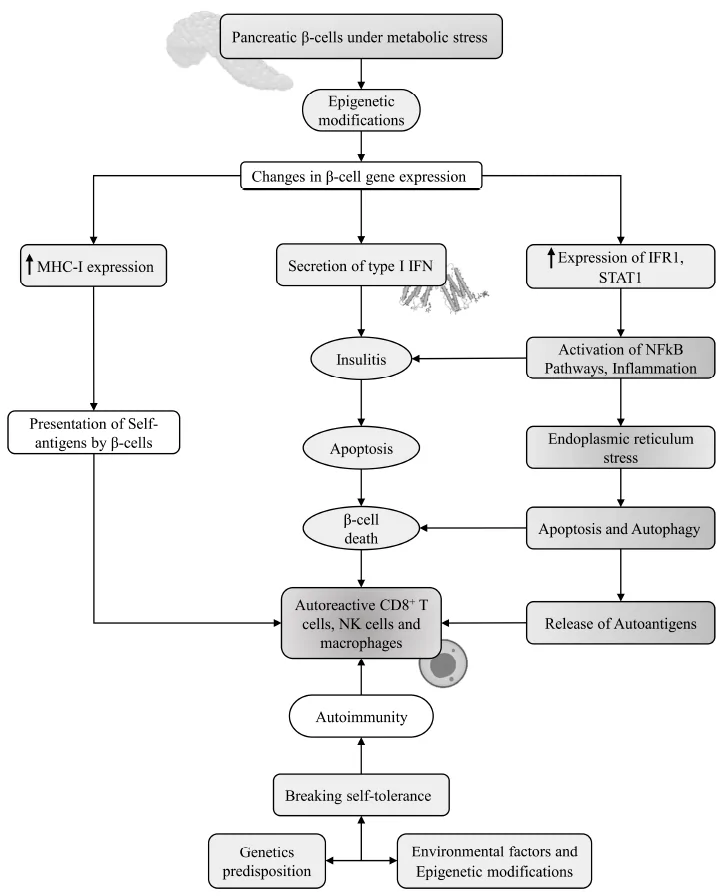
Representative scheme of the mechanisms that lead to the destruction of pancreatic β-cells. The β-cells of pancreatic islets, under conditions of metabolic stress, activate epigenetic modifications that promote profound changes in their gene expression, leading to the secretion of cytokines, mainly type 1 interferons (IFN-1), and the overexpression of MHC-I, increasing self-antigen presentation by β-cells. This also increases the expression of transcription factors STAT1, IRF1, and activation of the NF-κB pathway, generating oxidative stress in the endoplasmic and mitochondrial reticulum. Together, these factors can lead to insulitis and result in β-cell death through apoptosis and autophagy, releasing self-antigens from these cells. Conversely, when individuals with a genetic predisposition are exposed to environmental factors and epigenetic modifications, they may experience dysregulation of the immune system, resulting in a breakdown of self-tolerance and the development of autoimmunity. This leads to the production of T and B cells autoreactive to self-antigens released by β-cells, presented in the context of MHC-II. When CD4+ cells encounter these antigens, they become activated and differentiate into various Th cell phenotypes, which, in turn, activate B cells to differentiate into plasma cells that secrete autoantibodies against β-cells. Additionally, CD8+ T cells, NK cells, and autoreactive macrophages recognize and destroy β-cells by presenting these self-antigens in the context of MHC-I. Thus, β- cells subjected to excessive metabolic stress activate mechanisms that lead to their demise, releasing more self-antigens upon death, thereby activating further self-reactive immune cells. This creates a positive feedback loop resulting in the destruction of β-cells. Sequential arrows indicate key steps in the immunopathogenic cascade from β-cell stress to immune-mediated destruction. Intrinsic (β-cell-centered) and extrinsic (immune-mediated) factors interact dynamically, reflecting the interaction between genetic susceptibility, environmental exposures, and epigenetic remodeling.

**Fig. (2):**
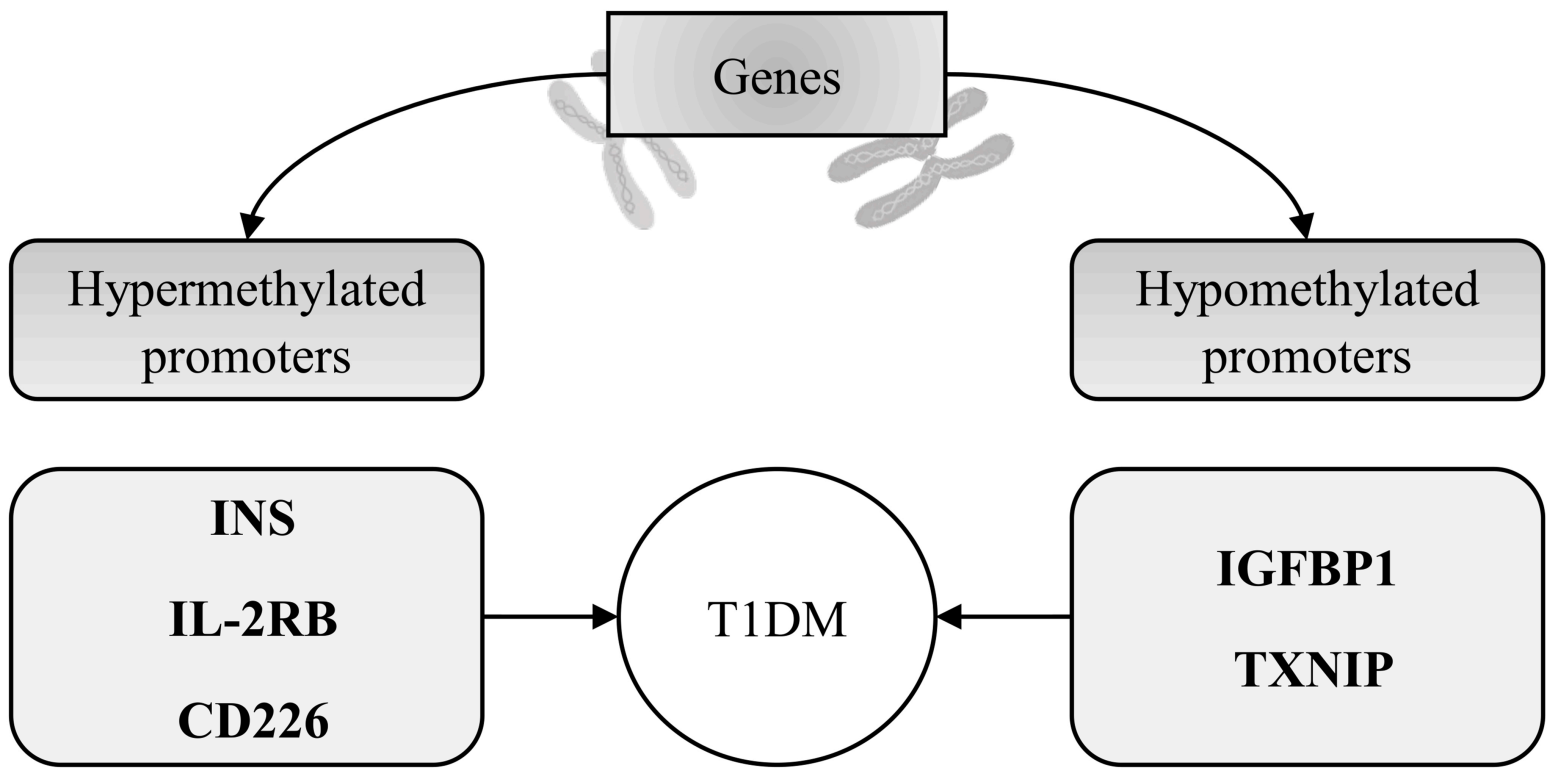
DNA methylation profiles of some genes associated with type 1 diabetes. Genes with hypermethylated promoters are silenced or expressed at very low levels, while genes with hypomethylated promoters are expressed at high levels. The INS gene encodes insulin, so its silencing leads to T1DM. The IL-2RB gene encodes the interleukin 2 receptor, which plays a role in the activation of Treg cells, protecting β- cells; therefore, its silencing contributes to the destruction of these cells. The CD226 gene regulates self-reactive immune cells that destroy β-cells. Thus, its absence impairs the action of Treg cells. The product of IGFBP1 binds to insulin-like growth factor, decreasing hormone production and contributing to disease development. The product of the TXNIP gene regulates β-cell functions, but when upregulated under stress conditions, it can induce apoptosis in these cells. Promoter methylation status directly influences gene expression related to immune tolerance, β-cell survival, and glucose metabolism. Arrows indicate whether gene expression is upregulated or downregulated, highlighting their impact on T1DM pathogenesis. These genes represent key epigenetic targets with potential for biomarker discovery and therapeutic intervention.

**Table 1:** List of Upregulated MicroRNAs in different stages of type 1 diabetes, their potential targets, and their respective functions.

**Type**	**Stage of the Disease**	**Target Genes or Signaling Pathways / Function**
miR-144-5p	Early disease	Target glycosaminoglycan synthesis pathways and PI3/AKT, MAPK, and WNT pathways.
miR-222-3p		
miR-345-5p		
miR-454-5p		
miR-let-7e-5p	Early disease	Target PI3K/Akt, MAPK, and WNT pathways, which are negatively correlated with glycated hemoglobin.
miR-324-5p		
miR-101-3p	Early disease	Target HGF/C-Met, Ephrin, and STAT3 pathways.
miR-200a-3p	Early disease	The target gene PRPP is related to metabolic pathways.
miR-346	Long-standing disease	
miR-26b-5p	Phase not defined	The target gene IGF1R is related to insulin signaling and apoptotic events.
miR-100-5p		
miR-143-3p		
miR-1-3p	Early disease	Target gene KEGG is involved in pathways related to endocrine, inflammatory, and immunological disorders.
miR-101-3p	Long-standing disease	Target AKT1, VEGFA, and IGF1 pathways related to activation of β-cell metabolism, which can lead to apoptosis of these cells, angiogenesis, and insulin signaling.
miR-135a-5p		
miR-143-3p		
miR-223-3p		
miR-410-3p		

**Table 2:** List of Downregulated Micro-RNAs in different stages of type 1 diabetes, their potential targets, and their respective functions.

**Type**	**Stage of the Disease**	**Target Genes or Signaling Pathways / Function**
mirR-154	Early disease	Target glycosaminoglycan synthesis pathways and target PI3K/Akt, MAPK and WNT pathways. these were positively correlated with glycated hemoglobin.
miR-490-5p		
miR-630		
miR-639		
miR-675b		
miR-146a-5p	Long-standing disease	The AKT1/ERK pathway was inversely associated with chronic complications.
miR-16-3P	All stages of disease	GADD45 is a target gene involved in cell cycle control, senescence, and autophagy pathways.
miR-495-3p	Long-standing disease	The target genes are related to the AKT1, VEGF, and IGF-1 pathways.

## LIST OF ABBREVIATIONS

MHC: Major Histocompatibility Complex
GAD: Glutamic Acid Decarboxylase
DM1: Type 1 Diabetes
Tregs: Regulatory T Cells
DN: Diabetic Nephropathy
NGT: Normal Glucose Tolerance
GWAS: Genome-wide Association Study
T1DM: Type 1 Diabetes Mellitus
